# Less invasive treatment of sleep-disordered breathing in children with syndromic craniosynostosis

**DOI:** 10.1186/s13023-018-0808-4

**Published:** 2018-04-23

**Authors:** Silvia Müller-Hagedorn, Cornelia Wiechers, Jörg Arand, Wolfgang Buchenau, Margit Bacher, Michael Krimmel, Siegmar Reinert, Christian F. Poets

**Affiliations:** 1 Department of Orthodontics, Rostock University Hospital, Rostock, Germany; 2 Department of Orthodontics, Tübingen University Hospital, Tübingen, Germany; 3 Center for Cleft Palate & Craniofacial Malformations, Tübingen University Hospital, Tübingen, Germany; 4 Department of Neonatology, Tübingen University Hospital, Calwerstrasse 7, 72076 Tuebingen, Germany; 5 BIP - Orthodontic Practice, Tübingen, Germany; 6 Department of Maxillofacial Surgery, Tübingen University Hospital, Tübingen, Germany

**Keywords:** Upper airway obstruction, Syndromic craniosynostosis, Palatal plates, Midface hypoplasia, Orthodontic treatment

## Abstract

**Background:**

Infants and children with syndromic craniosynostosis (SCS), such as Apert-, Crouzon- or Pfeiffer syndrome, are prone to sleep disordered breathing (SDB) including obstructive sleep apnea and upper airway resistance syndrome (OSAS, UARS), potentially leading to tracheostomy. We modified the Tübingen Palatal Plate (TPP), an oral appliance with a velar extension effectively treating airway obstruction in Robin sequence, by attaching a tube to its velar extension to bridge the narrow pharyngeal airway in SCS patients. Here, we evaluated this treatment concept.

**Methods:**

Our hospital’s electronic patient files were searched for all children with a diagnosis of SCS admitted between 01/01/2004 and 31/12/2016. Children with isolated craniosynostosis were excluded. OSAS was defined as a mixed-obstructive apnea-hypopnea index (MOAHI) > 1, and UARS as more than 1 episode with nasal flow limitation/h, but absent OSAS. Children with a diagnosis of OSAS received the TPP and fiberoptic nasopharyngoscopy to assess the type of obstruction and to adjust the plate. Growth and weight gain, determined as standard deviation scores, were also evaluated before and during treatment.

**Results:**

Of 34 patients included, 24 presented with SDB (19 OSAS, 5 UARS) and 27 had midface hypoplasia. Proportions of SDB were 78% in those with, and 22% in those without midface hypoplasia. In the OSAS group (*n* = 19), 13 patients were treated with palatal plates, with the remaining receiving continuous positive airway pressure, midface surgery or tracheal intubation. The MOAHI decreased across all children receiving palatal plate treatment from 14.6 (range 0.0–50.7) at admission to 0.9 (range 0.0–3.5) at discharge (*p* = 0.002). SDS for weight and body length also improved (*p* < 0.05 for weight and *p* = 0.05 for body length). Only one child required tracheostomy.

**Conclusion:**

Treatment of upper airway obstruction by a modified TPP in these children with SCS was shown to be mostly effective and safe. If confirmed in larger prospective studies, it may help to avoid more invasive interventions.

## Background

Infants and children with syndromic craniosynostosis (SCS), such as Apert-, Crouzon- or Pfeiffer syndrome, are prone to obstructive sleep apnea syndrome (OSAS), with prevalences ranging from 40 to 80% [1–3]. In some cases, OSAS is so severe that it leads to early tracheostomy placement [4, 5].

**Fig. 1:**
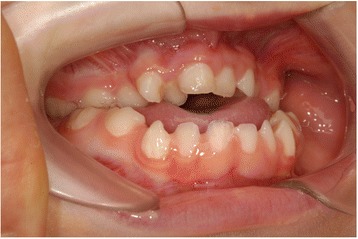
Intraoral photo of patient with Apert syndrome and midface hypoplasia (photo shown with parental permission)

Mutations in the fibroblast growth factor receptor (FGFR) are responsible for most SCS [6]. The primary anomaly involves the cranial base [7] and midface, where a premature fusion of craniofacial sutures leads to midface hypoplasia and retrusion. This midface hypoplasia is caused by a lack of sutural growth and an abnormal remodeling pattern resulting in a deficient maxilla that is small in all 3 planes (Fig. 1) [8]. This deficiency is aggravated during growth [9].

Endoscopically, SCS is characterized by multilevel airway obstruction with a reduced naso- and oropharyngeal airway space. The former is due to midface hypoplasia, the latter due to a lower tongue position narrowing the space between the tongue and the pharyngeal wall [10]. In addition, there may be central sleep apnea resulting from restricted brain growth, increased intracranial pressure and Arnold-Chiari malformation [11, 12]. Both, central and obstructive apnea may lead to neurocognitive impairment, behavioral difficulties, failure to thrive, pulmonary hypertension, congestive heart failure and even sudden death [13].

Many treatment options have been proposed, including adenotonsillectomy [14, 15], nasal positive pressure support (via continuous positive airway pressure (CPAP)) [16] or high-flow nasal cannula), nasopharyngeal airway [17], midface advancement [18, 19] and tracheostomy [5, 20]. Up to now, however, there is no consensus on the optimal approach to airway management in these patients.

**Fig. 2:**
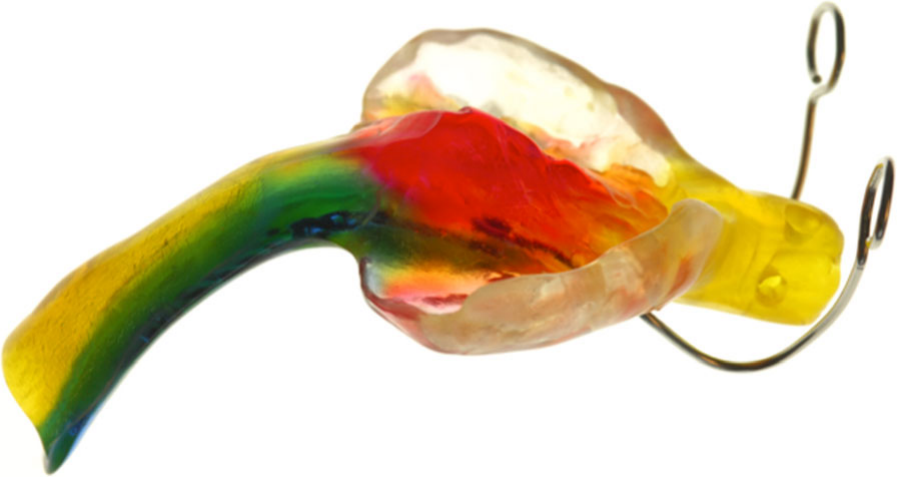
Modified Tübingen Palatal Plate

In our center, an oral appliance, a modified Tübingen Palatal Plate (TPP), in combination with Manual Orofacial Therapy (MOT) according to Castillo-Morales® [21], is used as a first line treatment for children with SCS. This modified TPP consists of a palatal plate with a velar extension and a tube attached to it (Fig. 2). The velar extension shifts the tongue into a more anterior position, the tube functions as an artificial airway to release the UAO. This treatment concept has been successfully evaluated in patients with isolated [22–24] and syndromic [25] Robin sequence, but little is known whether it is equally effective in children with SCS.

The aim of this study was to determine the prevalence of SDB in infants and children with SCS and to evaluate our treatment approach in this patient group. Because of their more complex anatomic disturbances a modification to the original TPP was often necessary (see below).

## Methods

### Patients

The hospital’s electronic database was searched for all children with a diagnosis of SCS admitted between 01/01/2004 and 31/12/2016. Children with isolated craniosynostosis were excluded.

### Data collection

The following clinical data were collected: underlying diagnoses, age, gender, type of cleft (if any), presence of choanal stenosis or midface hypoplasia, different treatment modalities and any treatment prior to admission to our center. Children undergoing TPP treatment had sleep studies performed prior to treatment onset and after each (re-)fitting of the plate. All other children had sleep studies performed at least once/year.

### Sleep studies

Sleep studies were performed using a computerized system (Embla N 7000, MedCare, Reykjavik, Iceland). The study montage comprised the following channels and sensors: chest and abdominal wall movements (respiratory inductive plethysmography, MedCare), nasal pressure and linearized nasal airflow (nasal prongs and built-in pressure transducer, MedCare), pulse oximeter saturation (SpO_2_) and pulse waveform (Radical, Masimo Inc., Irvine, USA), electrocardiogram (MedCare), and digital video via infrared camera (Panasonic; Tokyo, Japan). Recordings commenced in the evening and lasted for at least 8 h; all infants were studied in the supine position. Recordings were terminated if more than 3 desaturations to < 60% SpO_2_ occurred, and not attempted if upper airway obstruction prior to fitting the palatal plate was so severe that it could only be managed by endotracheal intubation.

All recordings were manually analyzed for the presence of respiratory events using slightly modified standard criteria [26] as used in our previous work [22–25]. In brief, total sleep time (TST) was determined from the first 10-min epoch without movement artifact or a distorted pulse waveform to the last such 10-min epoch; recordings comprising less than 3 h of TST were excluded. An apnea was scored if (i) the amplitude of the nasal airflow fell to < 20% of the average amplitude of the two preceding breaths, (ii) no airflow was detected at the mouth, and (iii) the event comprised at least two breath cycles (i.e. approximately 3–4 s). An obstructive apnea (OA) was scored if (i) the above criteria for apnea were fulfilled and (ii) out-of-phase movements of the chest and abdomen were present. A central apnea was scored if (i) criteria for apnea were fulfilled and (ii) no chest and abdominal wall movements were present. Mixed apneas were defined as those with both a central and an obstructive component, each lasting at least two breath cycles. In neonates and infants a mixed obstructive apnea index (MOAI) was calculated as the sum of mixed apneas plus OA per hour of TST. In children older than 12 months, hypopneas were also scored and the mixed-obstructive apnea-hypopnea index (MOAHI) determined: A hypopnea was scored if the nasal flow amplitude dropped by ≥30% of the pre-event amplitude lasting for at least 2 breaths and accompanied by a ≥ 3% desaturation. OSAS was defined as MOAI > 1 in infants and MOAHI > 1 in children older than 12 months (therefore denoted MOA(H)I in the following), and UARS [27] as more than 1 episode with nasal flow limitation/h, but without meeting OSAS criteria.

Desaturation events were visually confirmed to exclude spuriously low values. Events with a distorted pulse waveform signal within 7 s prior to their onset were considered artefactual and excluded. The number of desaturation events to < 80% SpO_2_ was counted and expressed as desaturation index, defined as events per hour of TST (DI_80_).

Children who had no sleep study performed because their upper airway obstruction had been so severe that they had arrived at our department already with an ET tube or tracheostomy in place were arbitrarily (and conservatively) assigned a MOA(H)I of 30 and a DI_80_ of 3 for statistical analysis of their sleep study results.

### Treatment protocol

After diagnosis, infants were admitted and monitored in the neonatal intensive care unit where they also underwent a baseline cardiorespiratory sleep study and fiberoptic nasopharyngoscopy without sedation to assess the type and localization of the UAO. This endoscopy usually took only 4–5 min. Children > 1 year were admitted to our pediatric sleep laboratory and fitting of the modified TPP was undertaken in the outpatient clinic.

Next, children had a maxillary imprint taken with a custom-made impression tray using alginate (Tetrachrom-Super-Alginat, ISO 1563, Klasse B, Typ I, Kaniedenta, Herford, Germany). This imprint covered the entire hard palate, the alveolar ridges and the vestibule. This procedure was carried out in the neonatal intermediate care unit under cardiorespiratory monitoring without sedation, but with a nasopharyngeal airway in place and in the presence of an experienced neonatologist. Older children had their imprints taken in the department of orthodontics without monitoring and nasopharyngeal airway in place. Then a plaster cast was produced using high precision dental plaster (Girodur Type IV, Synthetic Superhard Stone Plaster for Sectioned and Master Models DIN EN 26873, white, Girrbach Dental GmbH, Pforzheim, Germany). Using this cast, appliances were made from hard acrylic (autopolymerizing methylmethacrylate, Orthocryl, Dentaurum, Pforzheim, Germany). The TPP consisted of a palatal part that covered the hard palate and the cleft as well as the alveolar ridges and a velar extension of approximately 3 cm in length. The shape of the velar extension was modeled from dental wax and attached dorsally to the plaster cast. The length and the angle of the extension were chosen so that it was adjacent to the dorsum of the tongue. It was manufactured using a blue color to facilitate endoscopic evaluation in situ. After polymerization this prototype was polished using standard techniques.

Once a prototype of the plate was ready, infants had a repeat endoscopy to adjust the length and angle of the velar extension. The tip of the extension descended down to the vallecula epiglottica and the angulation was responsible for the anterior shifting of the base of the tongue and erection of the epiglottis, thereby widening the airway. Next, a tube made from hard acrylic was attached to the velar extension of the plate to form an artificial airway, followed by a repeat endoscopy with the endoscope introduced through this airway. If the airway appeared endoscopically and clinically open, the prototype was finished and a strengthening wire incorporated into the extension to safeguard the device against mechanical failure (Fig. 2). Two days later, its effectiveness was assessed by a second sleep study. If this sleep study still showed a MOA(H)I > 1, the plate was modified. Treatment in infants also comprised appropriate feeding techniques (finger feeding and Playtex Drop-Ins®, Playtex Products, Edgewell, North Bergen, NY, USA) and an orofacial stimulation therapy according to Castillo Morales®.

In infants and at the beginning of treatment, appliances were worn continuously and only removed for cleaning purposes. After 3–4 months of continuous treatment and in older children, the TPP was often only applied at nighttime. It was held in situ by adhesion and suction, by an adhesive cream (Blend-a-dent Super Haftcreme, Procter & Gamble, Schwalbach, Germany), and by extra-oral bows attached to the plate and fixed on the face with adhesive tape (Steri-Strip, 3 M Health Care, St. Paul, MN). Fitting of the plate was regularly controlled by the nursing staff. If the palatal part became too small, a new TPP was produced and fitted. After discharge, patients were seen at 6–8 weekly intervals at the orthodontic outpatient clinic; the next sleep study was performed 3 months after discharge. In general, new plates became necessary if a notch appeared on the alveolar ridges or sleep study results deteriorated again, which was often after approximately 3–6 months. In older children new plates became necessary with tooth eruption and skeletal growth.

### Statistical analysis

Results are reported as median and range. Comparisons between sleep study results were done using software (Statistical Package for the Social Science, Version 18, IBM, New York, USA). For the analysis of sleep parameters, the Wilcoxon signed rank test was used; correlations were assessed by Spearman’s rank correlation coefficient.

### Ethics

In Germany, retrospective audits of own patient data, aimed at quality improvement, do not require ethical approval.

## Results

We identified 34 patients (17 females) with SCS referred to our center: In 30 cases a well defined syndrome such as Apert, Crouzon, Pfeiffer, Saethre Chotzen or Cole Carpenter could be identified and in the other 4 cases an as yet unidentified or extremely rare SCS. At the time of admission or first outpatient visit, patients were between 1 day and 13 years old; most (*n* = 21) were infants, 4 toddlers, 4 preschool children; only 5 were already attending school. Two patients died of severe complications of their underlying Pfeiffer syndrome, unrelated to their respiratory condition.

Eight children presented with a cleft palate, 5 of them were diagnosed as Apert syndrome, one as Pfeiffer syndrome and two with an as yet unidentified craniosynostosis syndrome. Two children presented with choanal stenosis, one had Apert-, the other Crouzon syndrome. Out of 34 the patients included, those with an identified SCS (*n* = 30) were classified according to the presence or absence of midface hypoplasia (Table 1). There were 25 children with midface hypoplasia (Apert, Crouzon and Pfeiffer syndrome), and 5 without (Saethre Chotzen, Cole Carpenter syndrome).

**Table 1:** Diagnoses found and prevalence of obstructive sleep apnea syndrome (OSAS)/upper airway resistance syndrome (UARS), respectively

		No SDB	OSAS	UARS
SCS with midface hypoplasia (*n* = 25)	Pfeiffer syndrome (*n* = 4)	1	3	0
	Crouzon syndrome (*n* = 9)	2	5	2
	Apert syndrome (*n* = 12)	2	8	2
SCS without midface hypoplasia (*n* = 5)	Saethre Chotzen syndrome (*n* = 4) Cole Carpenter syndrome (*n* = 1)	4	0	1
Other syndromes associated with craniosynostosis (*n* = 4) (2 with midface hypoplasia)		1	3	0

Two children with midface hypoplasia were among those with a yet unidentified craniosynostosis syndrome.

### Sleep study results

Twenty-five patients had an initial sleep study comprising ≥3 h of TST, of which 14 had OSAS (14 with a MOA(H)I > 1, 10 of these with a MOA(H)I > 3), and 5 had UARS. In 5 children, no sleep study was possible because of clinically severe OSAS: one had received a tracheostomy prior to admission, and in 4, OSAS had been so severe that they had already been intubated at their referring hospitals. In the remaining 4 children, no sleep study was performed; none had clinical signs suggestive of OSAS.

In 4 children we were able to study the aggravation of UAO with age: 2 children presented with Pfeiffer-, 1 with Crouzon- and 1 with Apert syndrome. The latter had an initially normal sleep study (MOAI = 0) as a neonate, but a repeat sleep study, performed at 4 months of age, showed a MOAI of 14.6. In the remaining patients split night sleep studies were performed (with and without ongoing treatment). In all 4 patients, UAO worsened with age and a positive correlation between MOAHI and age was found in repeat split-night sleep studies (Spearman’s rank correlation coefficient r_s_ = 0.66, *p* = 0.004).

Prevalence of OSAS varied by diagnosis (Table 1). Considering the total group, OSAS prevalence was 56%, that of UARS 15%, together yielding an SDB prevalence of 71%. In identified SCS this proportion varied between 80% in those with Pfeiffer-, Crouzon- or Apert syndrome, and 20% in those with Saethre Chotzen- and Cole Carpenter syndrome. Considering all patients with SCS, prevalence of SDB was 78% in those with and 22% in those without midface hypoplasia.

Out of the 19 children presenting with manifest OSAS, 11 were infants, three were toddlers, one was of preschool and 4 of school age.

### Palatal plate treatment

**Fig. 3:**
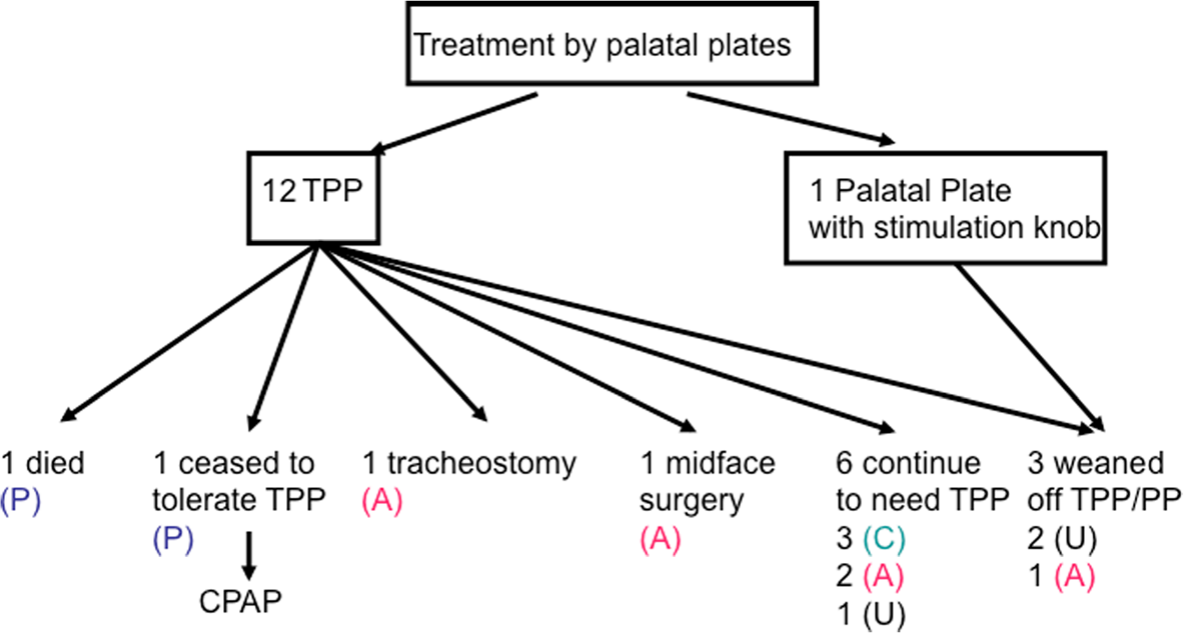
Flowchart of patients: P (Pfeiffer syndrome, A (Apert syndrome), C (Crouzon syndrome), U (undefined syndrome with craniosynostosis); PP (palatal plate), TPP (Tübingen Palatal Plate)

Thirteen children (68%) underwent palatal plate treatment for their OSAS (Fig. 3), all but one via a modified TPP that had a tube attached to the pharyngeal spur (Fig. 2); in 9 patients this treatment was started during their first year of life (Table 2). Median duration of hospital stay in these patients was 60 days (range 10–104).

**Table 2:** Treatment modalities in patients with OSAS by age at onset of therapy in our center (PP, palatal plates; TPP, Tübingen palatal plate; stim. knob, stimulation knob)

	Infants *n* = 11	Toddlers *n* = 3	Preschool children *n* = 1	School children *n* = 4
Palatal plates	9× PP • 8× TPP • 1× stim. knob	3× TPP		1× TPP (midface surgery later)
CPAP	1× CPAP (parental preference)		CPAP & adenotomy	2× CPAP (1× with midface surgery elsewhere but residual OSAS)
Midface surgery				3× midface surgery (1× after TPP)
Other	1× intubation (patient died 10 days after birth)			

### Sleep study results in patients with palatal plate treatment

These are shown in Table 3. One patient initially had no obstructive apnea, but severe oxygen desaturations (DI_80_ = 10), reaching down to 25% SpO_2_. During TPP treatment, sleep study results improved in all patients (Table 3).

**Table 3:** Sleep study results, shown as median (range). All palatal plates considered (12× TPP, 1× Palatal Plate with stimulation knob). Abbreviations: MOA(H)I, mixed-obstructive apnea (hypopnea) index; DI_80_, desaturation index to 80% pulse oximeter saturation

Parameter	Baseline sleep study	Sleep study with palatal plate	*p*-value
MOA(H)I	14.6 (0.0–50.7)	0.9 (0.0–3.5)	*P* = 0.002
DI_80_	1.0 (0.0–10)	0 (0.0–1.5)	*p* < 0.05

### Growth and weight gain

Weight and length were determined upon admission and discharge or at the next outpatient visit in all children receiving palatal plate treatment except for one toddler (*n* = 12) and expressed as standard deviation scores (SDS). Median SDS was < 0 for both parameters upon admission and decreased during treatment, although median SDS continued to be < 0 (Table 4). Differences were statistically significant for weight gain.

**Table 4:** Outcome of weight gain and growth in children with TPP treatment. Results are shown as SDS values (median and range); missing values for one patient

Parameter	Before treatment with TPP	At discharge/next outpatient visit	*p*-value
Weight gain	−2.08 (−4.02–0.36)	−1.50 (−6.0–2.09)	*p* < 0.05
Body length	− 1.27 (− 2.76–1.85)	−0.09 (− 2.35–1.34)	*p* = 0.05

### Outcome after TPP treatment

Out of the 9 patients with Apert-, Pfeiffer- and Crouzon syndrome, treatment could be discontinued in 1 child at age 11 years following surgical midface advancement. Of the remaining patients, 5 are continuing to wear their plates until midface surgery is planned at 12–14 years of age. One child (with Pfeiffer syndrome, see above) died, another also with Pfeiffer syndrome ceased to tolerate the TPP after 5.5 years of uneventful treatment and was continued on CPAP treatment. The remaining child underwent tracheostomy for severe respiratory distress due to recurrent mucus plugging of the tube attached to his palatal plate. (Fig. 3).

### Treatment of SDB using CPAP

**Fig. 4:**
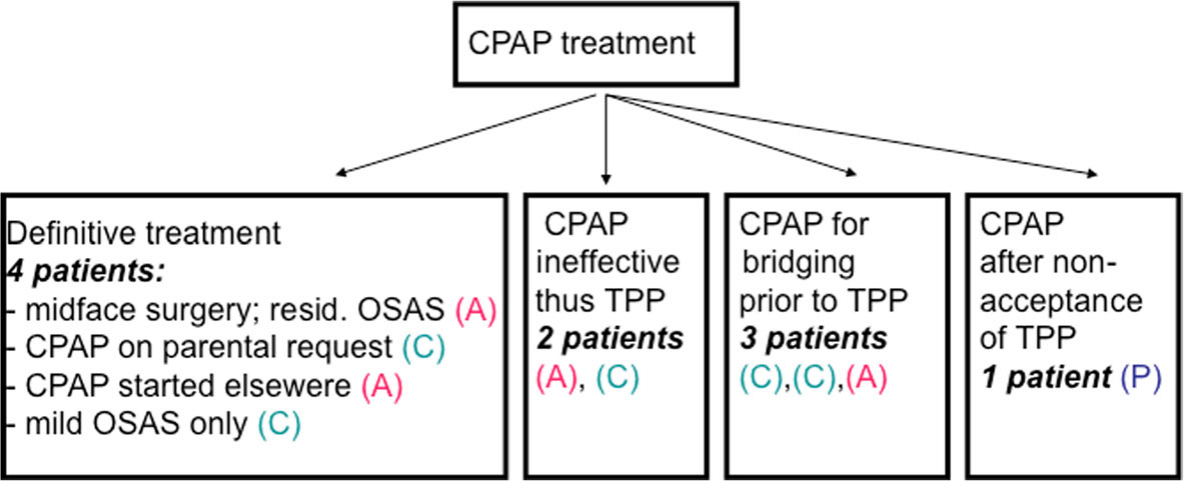
Flowchart of patients with CPAP treatment

Ten patients received respiratory support via CPAP for some time mostly using a full-face mask: they had already undergone midface distraction at an early age, but suffered from residual OSAS (1), used CPAP on parental request (1), had started CPAP at another hospital (1) or used CPAP only temporarily for mild OSAS (1). Five patients were successfully switched from CPAP (2 ineffective nasal CPAP, 3 CPAP as bridging treatment) to TPP. The remaining patient was that mentioned above who suddenly ceased to tolerate TPP treatment (Fig. 4).

### Treatment of UAO by surgical midface advancement

Three patients underwent surgical midface advancement at 6, 11 and 12 years, respectively: 2 had a tracheostomy in place, the third underwent surgery after 4.3 years of TPP treatment. Either treatment could be discontinued after surgery. The youngest patient re-developed OSAS 8 years following midface advancement and was then treated with CPAP.

## Discussion

This study confirms a high prevalence of SDB in patients with SCS [1, 28, 29] and presents results of a novel treatment approach to these patients. We found an overall SDB-prevalence of 71%, which increased to 78% if only those with midface hypoplasia were considered. The latter is caused by a growth deficit of the maxilla, which is not adequately displaced anteriorly relative to the anterior cranial base during growth and seems to be the most important etiologic factor for SDB in patients with SCS. Unlike Robin sequence, the mandible usually grows normally in SCS. Reitsma [30], in a cephalometric study, found an aggravation of midfacial underdevelopment with age, also reported by Reid [9], and even smaller sella-nasion-A point (SNA) angles in patients with Apert- than in those with Crouzon syndrome, suggesting that the former patients have a more severe midface deficiency. Accordingly, SDB became more severe with increasing age in our patients.

It is generally assumed that nasal obstruction in SCS is also secondary to midface hypoplasia leading to obstruction of the nasopharyngeal airway. In severe cases, these patients are mouth breathers so that their lower tongue position leads to a further decrease in oropharyngeal airway width [10]. This midface hypoplasia may explain the limited effectiveness of nasal CPAP in our patients and should be overcome by using full-face masks. However, there is a risk of disturbed midface development following longterm facial mask treatment [31–33], which has particularly to be avoided if the maxilla is already hypoplastic anyway.

Children with SDB have a higher energy expenditure due to an increased work of breathing, potentially resulting in severe failure to thrive. This could be confirmed in our patient group. Median SDS values for weight and growth were below 0 prior to TPP treatment but improved with treatment.

Midface advancement with LeFort III osteotomy and distraction alleviates UAO and may be considered a causal treatment, but is preferably postponed until the early teenage years to avoid interference with facial growth. One of our patients underwent midface advancement at 6 years of age, but had OSA recurrence 8 years after surgery, supporting an approach to postpone midface surgery until reaching early teen age.

Our treatment concept consists of palatal plates with a tubular structure bridging the narrow airway. This treatment modality has been successfully evaluated in infants with isolated and syndromic Robin sequence [22, 25]. While it may be considered a causal treatment in the former, providing a growth incentive to the hypoplastic mandible, it may be considered only a symptomatic treatment in patients with SCS, being similar in effect to a nasopharyngeal airway, CPAP or tracheostomy. In SCS, only surgical midface advancement or a LeFort III osteotomy may be considered causal treatments, because only these improve upper airway dimensions; their effectiveness has also been confirmed in a meta-analysis [16].

Similar to previous work from our group [22–25], we performed polygraphic sleep studies, not full polysomnography, the gold standard in sleep medicine. This happened to minimize interference with sleep in these already compromised patients and because sleep and wakefulness can also be determined reliably using cardiorespiratory and video recordings [34].

Initially, we were able to treat all patients successfully with a modified TPP. In our experience, acceptance of the plate is easier the earlier treatment is started. Therefore, a trial of this treatment is currently offered to all children with OSAS and SCS admitted to our center. Older children may also accept TPP treatment, but use the plate only during sleep. Treatment is usually continued until midface surgery can be performed.

## Conclusion

Palatal plate treatment in children with SCS and OSAS was shown to be an effective, safe and only minimally invasive option. It helps to avoid, or at least postpone, more invasive interventions, but requires an interdisciplinary team consisting of pediatricians trained in nasopharyngeal endoscopy, orthodontists, pediatric sleep specialists and speech therapists familiar with orofacial regulation therapy. The nursing team is also of outstanding importance, not least to train parents in handling the plate. It has to be pointed out that this kind of treatment is more difficult to apply in patients with SCS than in those with isolated RS and often requires a longer initial hospital stay.

## Abbreviations

CPAP: Continuous positive airway pressure
DI_80_: Desaturation index to 80% pulse oximeter saturation
ET tube: Endotracheal tube
FGFR: Fibroblast growth factor receptor
HFNC: High-flow nasal cannula
MOAHI: Mixed obstructive apnea hypopnea index
MOAI: Mixed obstructive apnea index
MOT: Manual orofacial therapy
OA: Obstructive apnea
OSAS: Obstructive sleep apnea syndrome
PP: Palatal plate
SCS: Syndromic craniosynostosis
SDB: Sleep disordered breathing
SDS: Standard deviation score
SNA angle: Sella-nasion-A point angle
SpO_2_: Arterial oxygen saturation measured by pulse oximetry
Stim. knob: Stimulation knob
TPP: Tuebingen palatal plate
TST: Total sleep time
UAO: Upper airway obstruction
UARS: Upper airway resistance syndrome
